# The Prevalence of Autism Spectrum Disorders in Adult Psychiatric Inpatients: A Systematic Review

**DOI:** 10.2174/1745017901814010177

**Published:** 2018-08-29

**Authors:** Samuel Tromans, Verity Chester, Reza Kiani, Regi Alexander, Terry Brugha

**Affiliations:** 1 Department of Health Sciences, University of Leicester, Leicester, Leicestershire, United Kingdom; 2 Leicestershire Partnership NHS Trust, Leicester, Leicestershire, United Kingdom; 3 Priory Group, Norwich, Norfolk, United Kingdom; 4 Norwich Medical School, University of East Anglia, Norwich, United Kingdom; 5 Hertfordshire Partnership University NHS Foundation Trust, Broadland Clinic & Astley Court, Norwich, United Kingdom

**Keywords:** Autism, Asperger, Psychiatric, Inpatient, Intellectual, Disability, Adult

## Abstract

**Background:**

Whilst the prevalence of autism spectrum disorders in adults within the community setting is well-established, less is known about the prevalence among adults based within a psychiatric inpatient setting.

**Objective:**

To conduct a systematic literature review pertaining to the prevalence of autism spectrum disorders among the adult psychiatric inpatient population.

**Method:**

Eligibility criteria included: (a) investigation of the prevalence of autism spectrum disorders (b) adult psychiatric inpatient study population (c) published in English language. Electronic databases accessed included PubMed, Medline, CINAHL, PsycINFO and EMBASE. Additionally, the ancestry method was utilised for the references of eligible papers, as well as grey literature searches and consultation with experts in the field.

**Results:**

From the search, 4 studies were identified which satisfied the inclusion criteria, conducted in a variety of inpatient psychiatric settings, including secure forensic and intellectual disability units and a state psychiatric hospital. There were significant differences in methodological approaches, including the screening tests, diagnostic instruments and diagnostic criteria utilised. Autism spectrum disorder prevalence estimates varied considerably, from 2.4-9.9%.

**Conclusion:**

From the limited research data currently available, it appears that the prevalence of autism spectrum disorders is increased in inpatient psychiatric settings relative to the general population. There is a need for further high quality research in this patient group, to add to this limited evidence base, as well as in developing effective strategies to identify patients with a high likelihood of autism spectrum disorders within this setting.

## 1 INTRODUCTION

Autism Spectrum Disorders (ASD) describe a range of neurodevelopmental disorders characterised by difficulties in social interaction and communication, as well as restricted, stereotyped and repetitive behaviours [1]. ASD subtypes include autism, Asperger’s Syndrome (AS) and Pervasive Developmental Disorder Not Otherwise Specified (PDD-NOS); a further two PDD’s exist (Rett’s syndrome and childhood disintegrative disorder), though these are qualitatively distinct from the aforementioned subtypes, and are thus not considered further in this review [2]. Though originating in childhood, the debilitating effects of ASD persist into later life, leading to many individuals requiring high levels of support throughout their adult life [3]. Indeed, whilst most research has historically focussed on ASD in children, in recent years there has been an increasing focus on the clinical needs of adult patients [4]. Despite this, service provision for adults with ASD is still in its relative infancy [5]. An important component of this is ready access to diagnostic services for adults with suspected ASD, as diagnosis is essential in both understanding their needs and planning their subsequent care [6].

Community-based prevalence rates for autism in the general population show considerable variation across studies, though recent systematic reviews and large-scale epidemiological research estimate rates of between 0.7-1.1% [7, 8]. Additionally, the prevalence of autism is significantly higher in people with moderate to profound intellectual disability (ID); Brugha *et al.* [8] found a prevalence of 39.3% (95% confidence interval 31.0-48.4) for this patient group, compared to 1.0% (95% confidence interval 0.4-2.2) for those with mild ID. For these reasons, ASD represents a major global public health issue, responsible for over 111 Disability Adjusted Life Years (DALY’s) per 100,000 persons [7].

However, while there has been a focus on the prevalence of ASD in the community, its prevalence within an inpatient psychiatric setting is less well established. Mandell *et al.* [9] (2012) cited several reasons to support the suggestion that ASD may be overrepresented and underdiagnosed among adults within such a setting, including a lack of training among adult psychiatrists in conditions originating in childhood. Additionally, presence of comorbid psychiatric disorders that are more common in people with ASD could potentially complicate the diagnostic picture [9, 10], including depression [11, 12], bipolar disorder [13], anxiety disorders [14], schizophrenia [13, 15], attention deficit hyperactivity disorder [11, 14], alcohol and substance abuse [13], as well as ID [16, 17]. Such conditions could lead to instances of failure to identify ASD where present, as well as misattribution of ASD symptoms to other forms of mental disorder, such as schizophrenia [9, 15]. Finally, the diagnostic criteria for ASD have broadened in recent decades, in contrast to the narrowing of those for schizophrenia [9, 18-20].

Understanding the prevalence of ASD within an inpatient psychiatric setting has significant implications for resource allocation by healthcare providers, as this patient group could potentially represent a target for case identification. Identifying individuals with ASD will ensure that the clinical needs of such individuals are better understood, and taken into account in any subsequent treatment approach [15]. This could potentially lead to improved therapeutic outcomes in both the shorter (*e.g* reduced length of hospital stay) and longer terms (*e.g* reduced likelihood of readmission).

The purpose of this systematic review was to evaluate the current evidence pertaining to the prevalence of ASD in adults within an inpatient psychiatric setting. This is the first systematic review on this topic.

## 2 MATERIALS AND METHODS

### 2.1 Systematic Search Strategy

The PubMed, MedLine, CINAHL, PsycINFO and EMBASE databases were searched from their respective inceptions to 10^th^ December 2017. Titles and abstracts were searched for the following terms: (((autism spectrum disorders OR autis* OR Asperger* OR PDD OR PDDs OR PDD-NOS OR ASD OR ASDs OR pervasive development* disorder* OR Kanner*))) AND (((prevalen* OR screen* OR rate*))) AND (((adult))). Please note that autis* was used as a search term to cover titles and abstracts containing either ‘autism’ or ‘autistic’; prevalen* was used as a search term to cover titles and abstracts containing either ‘prevalence’ or ‘prevalent’. All titles and abstracts of articles that remained following removal of duplicates were screened against the inclusion criteria by two investigators (ST + VC). If they were considered to potentially satisfy inclusion criteria, full texts were accessed. In instances where there was uncertainty between ST and VC regarding the eligibility of an article, the final decision was made by RK. The ancestry method was utilised to identify additional studies within the references of eligible papers. Grey literature searches included Google Scholar and manual searches. Experts in the field were consulted to identify any additional published or unpublished data; for all articles identified* via *expert consultation, the full texts of the articles were assessed. Two separate searches were conducted by the local research design and library services, to find studies not identified by the above methods. The details of the search were registered on the PROSPERO database (Registration No. CRD42017084616).

### 2.2 Study Selection

Studies were included provided they satisfied all of the following criteria: (a) investigation of the prevalence of autism spectrum disorders within a psychiatric inpatient population (b) adult patients (≥ 16 years of age) (c) published in English language. Please note that a basic requirement for ‘investigation of the prevalence of autism spectrum disorders’ was that all participants within the study population were subjected to some form of autism-specific testing. Several studies were identified which simply reported the proportions of inpatients whom were identified as having autism through routine clinical practice; for the purposes of this review, these were not considered true prevalence studies and were excluded accordingly. Such studies would likely underestimate prevalence, as the diagnostic possibility of autism is not being proactively assessed using relevant assessment tools in all individuals within the study population.

### 2.3 Data Extraction

For each article, data was extracted pertaining to the year and location of the study, as well as the number of individuals enrolled, screened (where applicable) and undergoing full diagnostic assessment. The methods employed for such assessments were also obtained, as well as the ASD diagnostic criteria used. Where available, information was also extracted regarding the individuals included as well as excluded from the study, as this would impact on the generalisability of any resultant prevalence estimate. The proportion of male patients was also documented, as to determine whether the study proportion disproportionately represented one gender over another.

### 2.4 Data Synthesis

The prevalence results obtained were considered both with regard to overall findings for ASD prevalence across available studies, as well as specifically within ID, non-ID and forensic subgroups.

### 2.5 Quality Assessment

All articles that qualified for inclusion were stored by one investigator (ST) according to the 22-item STROBE checklist of cross-sectional studies [21], as all studies were of this type. No studies qualifying for inclusion were excluded based upon their STROBE score.

## 3 RESULTS

### 3.1 Study Characteristics

Database searches were conducted on 10^th^ December 2017. The database search yielded a total of n=7463 articles, including n=2885 articles in PubMed, n=1199 articles in MedLine, n=296 articles in CINAHL, n=1045 articles in PsycINFO and n=2038 articles in EMBASE. A total of n=80 additional records were identified through other techniques (ancestry method, grey literature searches and expert consultation). Following removal of duplicates, n=4237 articles remained for screening. Fig. (**1**) illustrates a PRISMA flow chart summary of the systematic search.

Upon abstract screening, a further 4126 articles were excluded for a variety of reasons, including focusing on research topics unrelated to Autism Spectrum Disorder (such as Atrial Septal Defect), failing to satisfy the search criteria (*e.g* studies on child populations, not measuring prevalence of Autism *etc*.), or not being published in English language. Thus, full texts of 111 articles were assessed, of which 4 qualified for inclusion. Of the included papers, 3 were identified on the initial database search, and 1* via *the ancestry method. Grey literature searches and liaison with experts failed to identify any additional studies satisfying inclusion criteria.

### 3.2 Findings of Included Studies

Table **1** summarises the details of all four included studies. Publication dates for such studies ranged from 1982 to 2012, and all were conducted in England or North America. The characteristics of the study samples varied markedly, including both state [9] and secure psychiatric hospitals [22, 23], as well as long-stay treatment facilities for individuals with ID [24]. Limited data were available on the mean age for many included studies (though all were conducted on adult populations), with mean age ranging from 42 to 52 years. For all included studies, the majority of the study population comprised of males, though this varied considerably from 61-100%. All included studies were found* via *the database searching process, with the exception of Hare *et al.* [23], identified through the ancestry method. No additional eligible articles were identified through either grey literature searches or consultation with experts in the field.

All but one of the included studies estimated the prevalence of ASD in general, though the methods of diagnosis varied considerably, from review of existing case notes, to using more standardised tools, such as the Disability Assessment Schedule (DAS) [32], Handicaps, Behaviours and Skills Schedule (HBS) [27], and the Diagnostic Interview for Genetics Studies (DIGS) [29] and Autism Diagnostic Inventory-Revised (ADI-R) [30]. In contrast, Scragg and Shah [22] estimated the prevalence of AS only, rather than all subtypes of ASD.

Prevalence estimates for ASD ranged from 4 to 9.9%, with Scragg and Shah [22] estimating a prevalence of 1.5% (or 2.3% when equivocal cases are included) for AS only.

### 3.3 Subgroup Analyses

Shah *et al.* [24], the only eligible study where the entire study population had ID, provided an ASD prevalence estimate of 4%. Mandell *et al*. [9] provided an estimate of 19.6% from the subgroup of patients in their study whom had ID.

Two studies involved a mixture of ID and non-ID individuals. Mandell *et al.* [9] estimated an overall ASD prevalence of 9.9% whereas Hare *et al.* [23] estimated 2.4%, increased to 4.8% when equivocal cases were taken into account.

Though none of the studies were conducted on non-ID patients only, Mandell *et al.* [9] provided prevalence data pertaining to the non-ID subgroup of their study population, which yielded a prevalence estimate of 5.3%.

It is important to note that Scragg and Shah [22] do not discuss the ID status of their study population in general, only providing details that none of the individuals diagnosed with AS had ID. The aforementioned studies conducted by Hare *et al.* [23] and Scragg and Shah [22] were both based in forensic settings.

### 3.4 Qualitative Summary of Relevant Excluded Studies

A brief report by Ferriter *et al.* [33] was excluded as it represented a summary of the same data covered in the more comprehensive eligible article by Hare *et al.* [23], with additional evaluation of the interrater reliability of the ASDASQ screening tool [26].

A study conducted by Turygin *et al.* [34] was excluded due to the study population not being subjected to some form of autism-specific testing, and rather the prevalence estimate of 9.9% (for a population of patients with ID within a residential treatment facility) being based on case note review and routine clinical assessment alone. There were several other studies excluded for this reason, such as Esan *et al.* [35], Gustafsson [36], and Lyall and Kelly [37], among others, further detailed in the supportive/supplementary material.

An article by Heinrich *et al.* [38] was not included due to the study population excluding patients whom had previously been assessed for ASD, as well as the patients representing a mix of inpatients and community-based individuals. Their study involved diagnostic assessment of 381 adults with ID (220 male) according to ICD-10 criteria. Diagnostic decisions were made as part of a multidisciplinary case conference involving review of all available diagnostic information obtained* via *standardised ASD tools, including the ADI-R [30], Autism Checklist (ACL) [39], Autism Diagnostic Observation Schedule (ADOS) [40], scale of Pervasive Developmental Disorder in Mentally Retarded Persons (PDD-MRS) [41] and Music-based Autism Diagnostics (MUSAD) [42]. A total of 92 patients (24.1% of the study population) were subsequently diagnosed with ASD.

The most frequent rationales for rejection of the remaining excluded articles for which full text assessments were undertaken included the study population being based in the community setting, the article reporting purely the proportion of autistic patients identified through routine clinical practice, the article representing a review rather than primary research, and the study population being comprised of people with strongly suspected or previously diagnosed autism. For further details pertaining to excluded studies, as well as the rationale for exclusion of all studies undergoing assessment of their full texts, please refer to the supportive/supplementary material.

## 4 DISCUSSION

In this systematic review we aimed to assess the current evidence relating to the prevalence of ASD among adults within inpatient psychiatric settings. There was a relative dearth of eligible studies, supporting the assertion that there is a need for further high quality research in order to provide more definitive evidence regarding the prevalence of ASD in inpatient psychiatric settings.

From this pool of eligible studies, we found wide ranging prevalence estimates, though there appears to be a general trend suggesting ASD is more prevalent in inpatient psychiatric settings relative to community populations. The reasons for such variance in estimates are likely manifold, as there is significant heterogeneity among the eligible studies, in terms of the characteristics of the patient populations studied, the study design, as well as the assessment tools and diagnostic criteria used.

With respect to study populations, it is essential that in studies involving a mixture of ID and non-ID patients, separate prevalence estimates are made, given the sizeable disparity in ASD prevalence estimates between this groups in community-based prevalence studies [7, 8, 4, 3]. As a result, the meaningfulness of prevalence estimate for a mixed ID/non-ID study populations is limited, unless separate data for both groups is also reported. Ideally, patients with ID should be further sub grouped according to severity of ID, as risk of ASD increases with increasing severity of ID [8].

There was limited evidence among eligible studies pertaining to inpatient prevalence among non-ID adult patients; however, the only eligible study by Mandell *et al.* [9] yielded a prevalence of 5.6%, much higher than widely accepted community prevalence estimates for this group [7]. Clearly more research in non-ID inpatient populations is required to discern whether this result is valid and generalizable.

Additionally, there were no eligible studies focussed on ASD prevalence among groups of patients with discrete mental disorders, such as schizophrenia. This is an important area for future research, as it would shed light on both the extent of ASD comorbidity with specific conditions, as well as further explore diagnostic overshadowing in this context.

The data on inpatients with ID showed significant variability in terms of prevalence estimates, ranging from 4-19.6%. Given the wide-ranging estimates from studies conducted over a considerable time period, it is impossible to assert with confidence whether this represents a significantly different prevalence than in patients with ID in the community setting [43].

All eligible studies were focussed on predominantly male study populations. Of the two studies reporting data on gender differences, one described non-statistically significant differences (p> 0.05) in ASD prevalence between males and females [9] and the other [23] reported a higher prevalence rate among males of those whom were subjected to diagnostic assessment, though did not comment on whether this was statistically significant. Further work needs to be done to determine whether there is a difference in ASD prevalence in the psychiatric inpatient setting, as well as more generally. There has been a long-held belief that ASD is more prevalent in males than females, particularly in those without ID [44], though it is being increasingly questioned whether this represents a ‘true difference’, or is attributable to other factors, such as ASD screening and diagnostic tests being insufficiently sensitive for females [45], and/or females possibly having a greater ability to mask their symptoms (so-called ‘camouflaging’) [46].

Based on the findings from the eligible studies, including those conducted in a forensic setting [22, 23], there appears to be a general trend suggestive of an increased prevalence of ASD within inpatient psychiatric settings. This data from forensic units could however be confounded by other factors, such as a lack of data on the number of individuals with ID in both studies. While it is conceivable that the clinical features present in ASD, such as abnormal social interaction and communication, could predispose someone to offending, there is limited evidence to support the notion that people with ASD are at any greater risk of committing crimes than the non-ASD population, with the possible exceptions of arson and sexual abuse [47]. Furthermore, even if there were an increased risk of offending behaviours in those with ASD, this would not necessarily manifest itself as an increased prevalence within a forensic psychiatric inpatient setting, as many individuals with ASD may remain undiagnosed, avoid conviction for their offences or be dealt with* via *the criminal justice system route.

Several studies employed a multiple-stage study design, involving use of a screening tool to determine those whom progress to more comprehensive diagnostic assessment. Such an approach has similarly been used in community-based ASD prevalence studies in both child [48, 49] and adult populations [50]. This is logistically sensible, as many of the ASD diagnostic assessment tools are informant dependent and resource intensive, requiring a considerable amount of time and specialist input [50]. For these reasons, a multiple-stage approach enables coverage of a larger patient population per unit of resources [51]. Of course, the value of such an approach is contingent on the validity and reliability of both the constituent screening and diagnostic tests used.

Diagnostic criteria can have a profound effect on the resultant prevalence estimate; as a community population-based study by Cooper *et al.* [43] demonstrated, where ASD prevalence among the same group of 1,023 individuals with ID varied from 2.0-4.4% depending on the diagnostic criteria used, and yielded a value of 7.5% for clinical diagnosis by a specialist, considered the gold standard. Also, the eligible studies were published across a period of 30 years and over that time the diagnostic criteria and overall concept of ASD as a clinical entity have broadened considerably [9, 20-22]

In conclusion, there is a clear need for robustly conducted epidemiological research pertaining to the prevalence of ASD among adults in psychiatric inpatient settings, as the current evidence base is lacking. Such research needs to carefully consider the assessment tools and diagnostic criteria used, as well as a study design supporting coverage of a large patient group. Additionally, the characteristics of the study population need to be appropriately detailed, with prevalence estimates for ID and non-ID groups and both genders as a bare minimum requirement. Adult psychiatric inpatient populations could indeed potentially provide a target for identifying those with ASD, though it is first essential to first establish whether ASD is truly significantly more prevalent in people within this group relative to the community setting.

## Figures and Tables

**Fig. (1):**
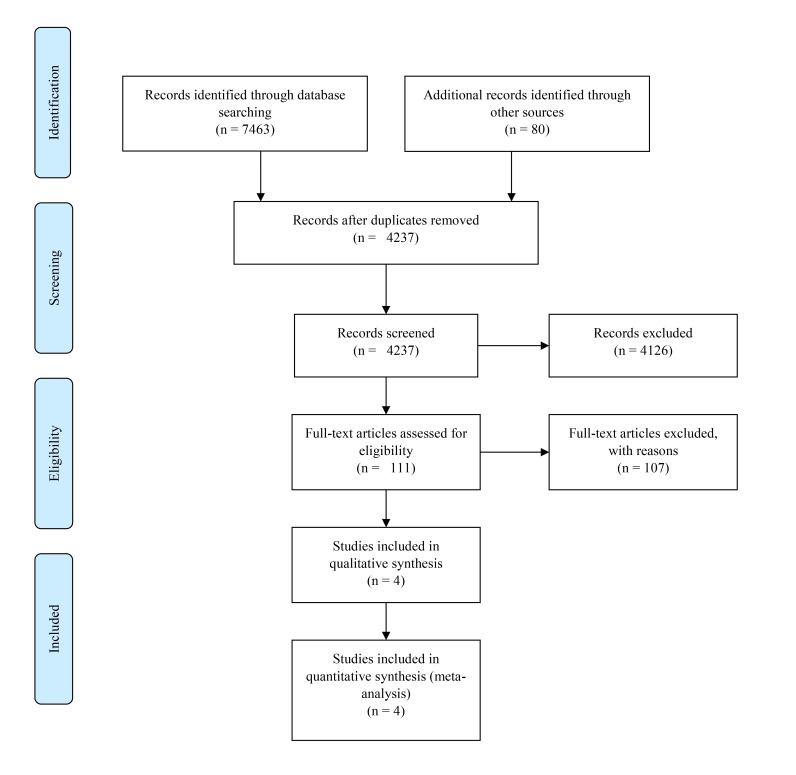
PRISMA flow diagram of systematic search [25].

**Table 1:** Summary of included studies.

**Author, Year, Location, Reference**	**Total N Enrolled in Study**	**N Screened**	**N Indicated for Diagnostic Assessment**	**N Subjected to Diagnostic Assessment**	**Details of Excluded Individuals**	**Male %**	**Mean Age in Years**	**Description of Inpatient Setting**	**Description of Study Population**	**Method of Screening**	**Method of Autism Diagnosis**	**Diagnostic Criteria Used**	**Prevalence Estimate**	**STROBE Score**
Hare *et al.* (1999), England, [23]	1305	1305	240	215	Patients on trial leave to other placements were excluded. Also, some were excluded from initial screening for administrative reasons of no clinical significance. 96% of the special hospital population was screened.	86% (185/215) of those subjected to diagnostic assessment. Data pertaining to the male % of the 1305 individuals originally screened is not available.	41.8 (Age range 20-77 years)	Three secure psychiatric hospitals	All adults. Mixture of non-ID and ID patient, though details regarding the relative proportions of each are not provided within the article.	Autism Spectrum Disorder in Adults Screening Questionnaire (ASDASQ)^1^ [26].	Handicaps, Behaviours and Skills (HBS) schedule [27] and case note analysis.	ICD-10 [1]	2.4% (31/1305), increased to 4.8% (62/1305) when equivocal cases are taken into account.	15/22
Mandell *et al.* (2012), USA, [9]	141	141 (though all patients were subjected to diagnostic assessment irrespective of screening score)	141	141	There were 348 residents within the hospital, so only 41% were screened (141/308). Researchers attempted to obtain consent from all residents; 89% of refusal were passive (*i.e.* patient unable to give consent). Thus, non-capacitous individuals were excluded.	75% (106/141)	52	State psychiatric hospital	Civilly committed patient. 32.6% (46/141) patients had ID. Age range not given.	Social Responsiveness Scale (SRS) [28].	Four step process: 1- Historical charts and electronic records reviewed; 2 – Diagnostic Interview for Genetics Studies (DIGS) [29] conducted for each patient; 3 – Autism Diagnostic Interview – Revised (ADI-R) [30] completed by research reliable clinicians; 4 – case conference review by two independent teams	DSM-IV^2^ [20]	Overall: 9.9% (14/141). ID subgroup: 19.6% (9/46). Non-ID subgroup: 5.3% (5/95).	16/22
Scragg and Shah (1994), England, [22]	392	392	17	17, though 6 refused to be meet the investigator	Female patient excluded, on basis that Asperger’s Syndrome is reporter to be much more common in males.	100% (392/392)	No data on age of study population, though the study population were adults.	Secure psychiatric hospital (Broadmoor, England)	Adult males. Unclear whether any patients in the study population had ID (details on IQ are only given for 9 patients whom met criteria for AS, none of whom had an IQ consistent with ID).	Examination of case notes	Two stages (after initial screening stage): 1 – Screening Schedule for Autistic Behaviour (Part of the HBS interview schedule) [27]; 2 – Interview by the investigator.	Gillberg and Gillberg (1989) [31] criteria	1.5% (6/392) (95% CI – 0.6 to 3.3%), increased to 2.3% with the addition of equivocal cases (However, this estimate was for Asperger’s syndrome only, rather than all forms of ASD).	12/22
Shah *et al.* (1982), England, [24]	761	n/a	761	761	Exclusion of 129 patients, due to being non-mobile – ‘their inability to walk unaided limited the possibility of their showing the behaviour pattern characteristic of classic Kanner’s syndrome.’	61% (468/761)	No data for mean age available. Youngest patients were 16 years of age.	Long stay ID hospital^3^	The entire study population had ID.	n/a	Disability Assessment Schedule [32]	Details not provided within the article.	4% (27 to 34/761). The precise number of participants being diagnosed with ASD is not provided within the article, though they report a prevalence of 4% from a study population of 761.	12/22

^1^ Not referred to as the ASDASQ within the article itself, as the tool was (at the time of the Hare et al article being published) an unnamed, unpublished screening tool (which was later named the ASDASQ). ^2^ Confirmed *via* correspondence with Dr David Mandell. ^3^ Referred to as a ‘mental handicap hospital’ within the source article.

## Footnotes

1 Not referred to as the ASDASQ within the article itself, as the tool was (at the time of the Hare et al article being published) an unnamed, unpublished screening tool (which was later named the ASDASQ).

2 Confirmed* via *correspondence with Dr David Mandell.

3 Referred to as a ‘mental handicap hospital’ within the source article.
