# Supplementary material for: The Prevalence of Autism Spectrum Disorders in Adult Psychiatric Inpatients: A Systematic Review
Source: Clin Pract Epidemiol Ment Health. 2018 Aug 29;14:177–87. doi: 10.2174/1745017901814010177 (PMC6118035; doi:10.2174/1745017901814010177)
Supplement: Supplementary file 1 [file CPEMH-14-177_SD1.pdf]

Send Orders for Reprints to reprints@benthamscience.ae

BENTHAM OPEN

CrossMark

Clinical Practice & Epidemiology in Mental Health

Supplementary Material

Content list available at: www.benthamopen.com/CPEMH/

DOI: 10.2174/1745017901814010177, 2018, *14*, i

The Prevalence of Autism Spectrum Disorders in Adult Psychiatric Inpatients: A Systematic Review

Samuel Tromans[1,2,*], Verity Chester[3,4], Reza Kiani[1,2], Regi Alexander[1,5] and Terry Brugha[1,2]

[1]*Department of Health Sciences, University of Leicester, Leicester, Leicestershire, United Kingdom*
[2]*Leicestershire Partnership NHS Trust, Leicester, Leicestershire, United Kingdom*
[3]*Priory Group, Norwich, Norfolk, United Kingdom*
[4]*Norwich Medical School, University of East Anglia, Norwich, United Kingdom*
[5]*Hertfordshire Partnership University NHS Foundation Trust, Broadland Clinic & Astley Court, Norwich, United Kingdom*

Received: April 25, 2018 Revised: May 22, 2018 Accepted: June 16, 2018

SUPPLEMENTARY TABLE

Summary Table	Database search	Ancestry method	Expert and grey literature	Overall
Total records identified	7463	18	62	7543
Total duplicates	3300	6	0	3306
Rejected immediately	4022	0	0	4022
Abstract reviewed then rejected	93	11	0	104
Full paper reviewed then rejected	45	0	62	107
Accepted	3	1	0	4

© 2018 Tromans *et al*.

This is an open access article distributed under the terms of the Creative Commons Attribution 4.0 International Public License (CC-BY 4.0), a copy of which is available at: https://creativecommons.org/licenses/by/4.0/legalcode. This license permits unrestricted use, distribution, and reproduction in any medium, provided the original author and source are credited.
